# A combined activation mechanism for the glucagon receptor

**DOI:** 10.1073/pnas.1921851117

**Published:** 2020-06-22

**Authors:** Giulio Mattedi, Silvia Acosta-Gutiérrez, Timothy Clark, Francesco Luigi Gervasio

**Affiliations:** ^a^Department of Chemistry, University College London, London WC1E 6BT, United Kingdom;; ^b^Computer-Chemistry Center, Department of Chemistry and Pharmacy, Friedrich-Alexander-University Erlangen-Nürnberg, Erlangen 91052, Germany;; ^c^Institute of Structural and Molecular Biology, University College London, London WC1E 6BT, United Kingdom;; ^d^Pharmaceutical Sciences, University of Geneva, Geneva CH-1211, Switzerland

**Keywords:** GPCR, glucagon receptor, activation, enhanced sampling

## Abstract

We report on a combined activation mechanism for a class B G-protein–coupled receptor (GPCR), the glucagon receptor. By computing the conformational free-energy landscape associated with the activation of the receptor–agonist complex and comparing it with that obtained with the ternary complex (receptor–agonist–G protein) we show that the agonist stabilizes the receptor in a preactivated complex, which is then fully activated upon binding of the G protein. The proposed mechanism contrasts with the generally assumed GPCR activation mechanism, which proceeds through an opening of the intracellular region allosterically elicited by the binding of the agonist. The mechanism found here is consistent with electron cryo-microscopy structural data and might be general for class B GPCRs. It also helps us to understand the mode of action of the numerous allosteric antagonists of this important drug target.

## Significance

Understanding the mechanisms of activation of G-protein–coupled receptors (GPCRs) is a major issue in biophysics and pharmacology. This is particularly true for peptide-activated class B receptors, which are more flexible and have been studied less than class A. Here, we combine simulations and free-energy landscape calculations to study the activation mechanism of the glucagon receptor, a prototypical class B GPCR. In contrast to previous conformational selection hypotheses, we find that both interactions with the peptide and the G protein are necessary to induce the transition to the active state. The results of this study not only contribute to a better understanding of GPCR activation mechanisms but will also aid in the future development of drugs targeting the glucagon receptor.

G-protein–coupled receptors (GPCRs) are the largest mammalian transmembrane receptor superfamily. Their variety and role in physiological pathways make them critical drug targets for numerous pathological conditions. Class B GPCRs, which bind peptide hormones such as calcitonin, parathyroid hormone, glucagon, and glucagon-like peptide 1 (GLP-1), are generally less well understood than their more common class A relatives.

The typically assumed GPCR activation mechanism, which is mainly based on data from class A receptors, posits that upon binding of an agonist on the extracellular side GPCRs undergo a large-scale conformational change resulting in the opening of a cavity in the intracellular side of the protein, whereupon G protein binds. Structural information about class B GPCRs is scarcer than for class A, yet available X-ray and electron cryo-microscopy (cryo-EM) structures indicate that this class undergoes a much more significant rearrangement upon activation than class A GPCRs, involving an extensive conformational change of the transmembrane helix (TM) 6 (1–12).

Glucagon receptor (GCGR) is a class B GPCR that mediates the glucagon-induced release of glucose from the liver into the bloodstream. It is being investigated as a potential target for the treatment of type 2 diabetes, complementing approaches that involve insulin signaling (13, 14).

A number of small molecules have been shown to interact with a transmembrane allosteric site, blocking the full activation of the glucagon receptor and glucagon-like peptide 1 receptor (GLP-1R) by “clamping” TM6 (15–17), highlighting the underlying complexity of the activation mechanism, and the need to understand the conformational dynamics associated with the activation of the receptor for the rational design of allosteric modulators.

Here we use molecular dynamics (MD) simulations and enhanced-sampling methods to compute the activation free-energy landscapes of the receptor in complex with glucagon and with both glucagon and the G protein. We elucidate the rearrangement of conserved motifs of the glucagon receptor that allows for the transmission of glucagon signaling to the intracellular side of the protein but does not lead to a fully active state. When we recalculate the free energy associated with the activation in conjunction with the G_α__s_ protein coupling, it becomes apparent that the fully active state is stabilized by the combined action of the extracellular and intracellular partners in inducing the conformational rearrangement of GCGR.

In this work superscripts to the residue numbers refer to the Wootten numbering scheme (18); additionally, the superscript “P” is used for glucagon residues and “G” for G_α__s_.

## Results and Discussion

### Glucagon Receptor Activation.

The activation free-energy landscape of glucagon receptor in complex with glucagon was calculated using parallel tempering well-tempered metadynamics (19, 20), a method that has been successfully used to compute free-energy landscapes of complex conformational rearrangements in various receptors, including GPCRs (21–25). The collective variables (CVs) used were *CV*_*Prog*_ and *CV*_*Dist*_, representing two linear combinations of the RMSD_Cα_ of TM6 to the conformation of inactive GCGR [Protein Data Bank (PDB) 5YQZ (26)] and to the active, closely related, GLP-1R [PDB 5VAI (27)]. GLP-1R was used as, at the time of the simulations, experimental models of active glucagon receptor were not available. During the peer review process a cryo-EM structure of active GCGR bound to G_s_ was released (4), providing experimental validation to our model. *CV*_*Prog*_ was calculated as the difference between the RMSD to the active and inactive structures, while *CV*_*Dist*_ was calculated as the sum between the two values. *CV*_*Prog*_ approximates the reaction coordinate: As it decreases, the receptor transitions from inactive-like to active-like conformations. *CV*_*Dist*_ is instead a measure of how far the system deviates from a linear interpolation between inactive and active states, allowing the exploration different activation pathways.

**Fig. 1.**
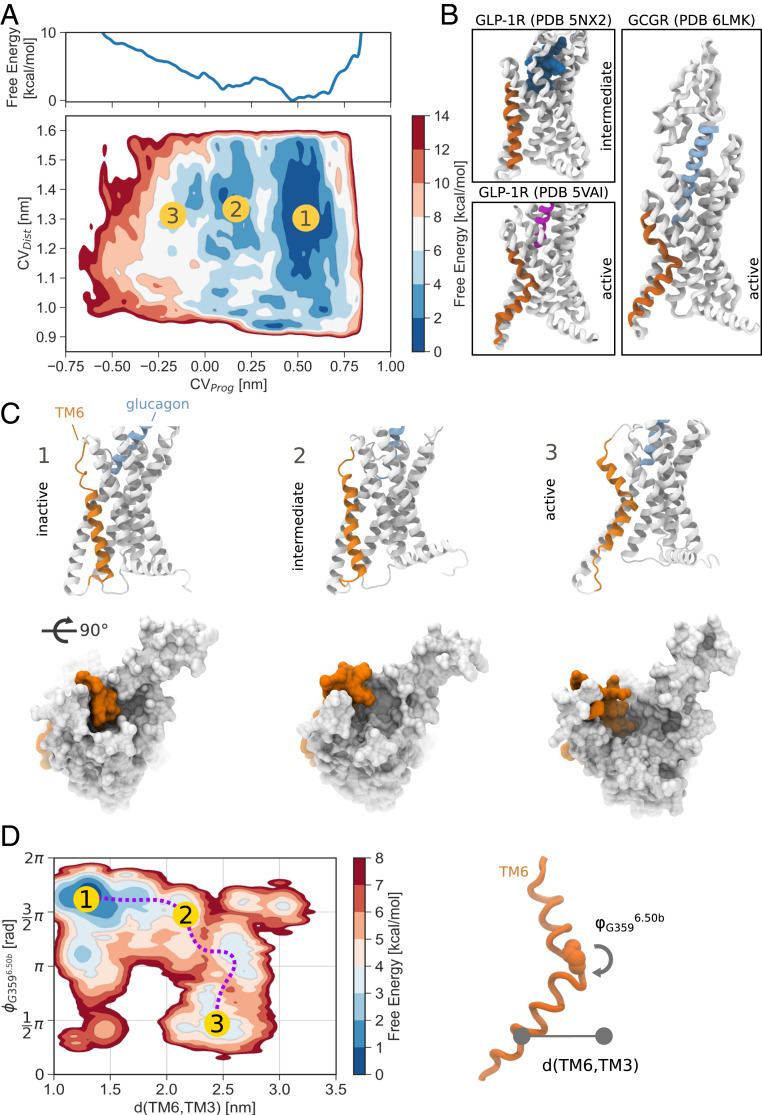
Activation free energy of glucagon receptor in complex with glucagon. (*A*) Activation free-energy landscape of glucagon receptor in the absence of G_α__s_. The marginal plot shows the projection of the free-energy surface onto the *CV*_*Prog*_ collective variable. (*B*) Intermediate GLP-1R [PDB 5NX2 (28)], active GLP-1R [PDB 5VAI (27)], and active GCGR [PDB 6LMK (4)]. (*C*) Representative conformations associated with the states indicated in the free-energy surface. (*D*) Reweighting of the free-energy landscape onto the centers of mass of the intracellular ends of TM6 and TM3 and the ϕ dihedral angle of G359^6.50b^. The activation of the receptor can be followed along the distance between TM6 and TM3 and the rearrangement of the ϕ dihedral of G359^6.50b^ of the *PxxG* motif.

The free-energy landscape shows three main minima corresponding to fully inactive, intermediate, and active conformations of the receptor (Fig. 1 *A* and *C*). The inactive state is associated with the lowest free energy and is therefore the most stable, while intermediate and active conformations are characterized by higher free-energy values. In the inactive state, TM6 adopts a fully helical conformation, close to that observed in the starting structure and X-ray and cryo-EM structures of other class B GPCRs (15, 16, 27) (Fig 1*C*, inactive). In this conformation the intracellular cavity found in active receptors is absent.

The intermediate state (Fig 1*C*, intermediate) is associated with a conformation of TM6 that resembles the one observed in thermostabilized GLP-1R bound to a peptide agonist (28) (Fig. 1*B*). The intracellular half of the helix is positioned about 0.4 nm away from the inactive conformation, extending away from the transmembrane domain (TMD). Yet, comparing the conformation of TM6 with that of X-ray and cryo-EM structures of active class B GPCRs (4–12), it is clear that the helix is not compatible with a fully active state, because of the absence of a sharp bend around the conserved *PxxG* motif of TM6 (P365^6.47b^-LL-G359^6.50b^ in glucagon receptor).

A higher-energy active state at *CV*_*Prog*_ ≈ −0.30 nm corresponds to a large conformational change of TM6 and TM5 (Fig 1*C*, active). A rearrangement of the backbone dihedral angles of the *PxxG* motif leads to the local unfolding of the region, bringing the angle formed by the top of TM6, the motif, and the bottom of the helix to around $110^{○}$. This allows TM6 to reach even farther away from the TMD, opening the intracellular cavity in which the G protein can bind.

### Rearrangement of Motifs and Networks.

**Fig. 2.**
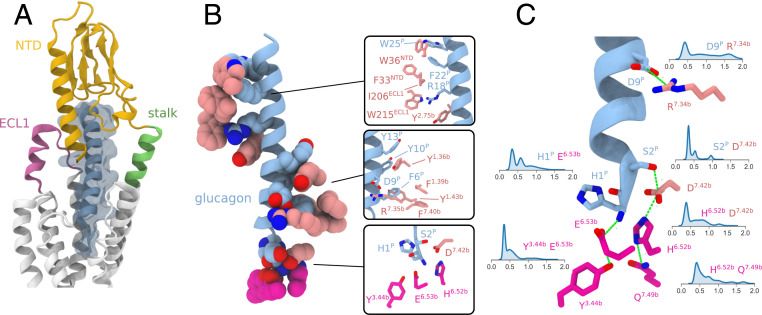
Interactions of glucagon with glucagon receptor. Shown are main interactions of the peptide with glucagon receptor in representative snapshots from the metadynamics simulation of the activation of glucagon receptor in absence of G_α__s_. (*A*) Overview of structural elements of GCGR interacting with the peptide. (*B*) Main interactions of glucagon with the TMD binding site and the NTD. (*C*) Key polar interactions involving the N-terminal region of the peptide and the TMD binding site and their distributions in the metadynamics simulation.

Throughout the simulation glucagon remained stably bound to the receptor. Extensive contacts with the N-terminal domain (NTD) and extracellular loop 1 (ECL1) confer remarkable stability to the bound peptide (Fig. 2*B*).

The N terminus of glucagon is hosted in the extracellular cavity of the TMD, with particular involvement of the extracellular ends of TM1, TM2, TM3, TM5, TM6, TM7, and ECL2. This region of the peptide is represented by a series of polar residues H1-SQG-T5^P^, while F6^P^ is hosted in a hydrophobic pocket lined by Y138^1.36b^, F141^1.39b^, Y145^1.43b^, and L386^7.43b^ (Fig. 2*B*). Interactions between the N terminus of glucagon and the host are fundamental for receptor activation (26).

**Fig. 3.**
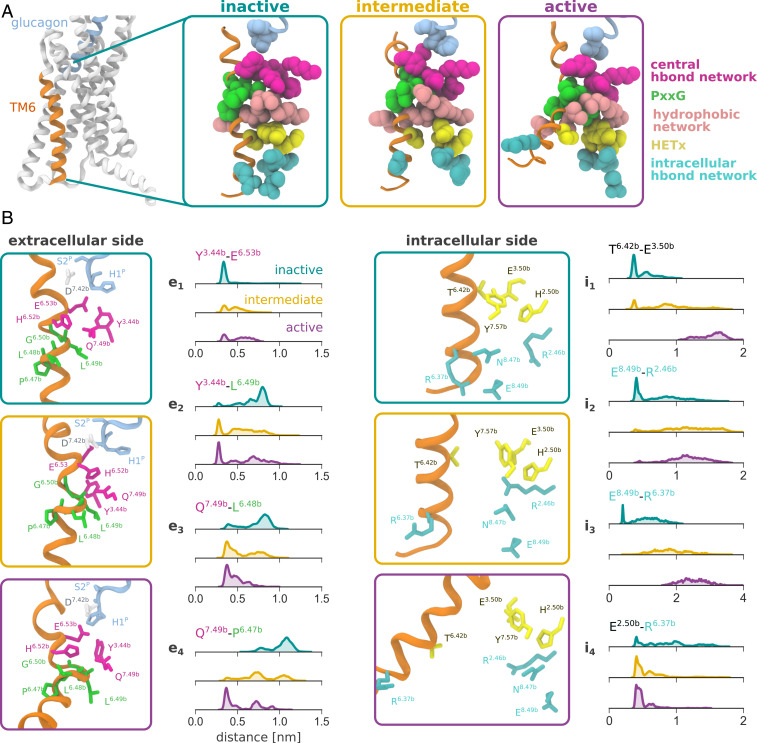
Rearrangement of main conserved TMD motifs and networks in inactive, intermediate, and active states of the receptor from the metadynamics simulation of activation of glucagon receptor in absence of G_α_. (*A*) Overview of the conformation of the receptor in the three states. (*B*) Distribution of the distances of key interactions in the states. The distances to P356^6.47b^, L357^6.48b^, and L358^6.49b^ were calculated using the backbone carbonyl oxygen atom, while for other residues heavy atoms of the side chains were used.

Below the binding site of the N terminus of the peptide is the central hydrogen bond network (2), represented by K187^2.60b^, N238^3.43b^, Y239^3.44b^, H361^6.52b^, E362^6.53b^, and Q392^7.49b^ (Fig. 3*A*). This network has been found to stabilize the inactive form of the receptor (2, 29) and has a crucial role in the function of GCGR (*SI Appendix*, Fig. S8). In the inactive state, glutamate E362^6.53b^ interacts with Y239^3.44b^ (Fig. 3*B*, e_1_). In our simulations glucagon, via the terminal backbone amine of H1^P^, forms a charged interaction with E362^6.53b^ (Fig. 2*C*) which can in turn allow for rearrangement of the tyrosine side chain for interaction with the backbone of L358^6.49b^ of the *PxxG* motif (Fig. 3*B*, e_2_).

The rearrangement of TM6 involves the conserved *PxxG* motif of the helix. This region, located next to the central hydrogen bond network, acts as a hinge for the conformational change of TM6. In particular, the flexible backbone of G359^6.50b^ undergoes the most significant rotation (*SI Appendix*, Fig. S9*C*). The presence of glycine and proline residues reduces the helical propensity of the motif, providing an effective weak point for TM6 bending. The importance of the two residues for the conformational rearrangement is confirmed by mutagenesis experiments that resulted in more rigid and easier to crystallize structures of numerous class B GPCRs (16, 27, 28).

The exposure of the backbone of the *PxxG* motif during the activation process results in the formation of a series of polar interactions with the central hydrogen bond network. Starting from the inactive structure, where a fully helical conformation is observed, partial rearrangement in the intermediate state allows for interaction between Y239^3.44b^ and L358^6.49b^, and Q392^7.49b^ and L357^6.48b^ (Fig. 3*B*, e_2_ and e_3_). The availability of Q392^7.49b^for hydrogen bonding with the *PxxG* motif is influenced by contacts with H361^6.52b^ and the interaction of the histidine with D385^7.42b^. The interaction between the histidine and aspartate side chains is destabilized in intermediate and active states, promoting the rearrangement of Q392^7.49b^ for interaction with the *PxxG* motif. Upon complete rearrangement in the active state, the backbone of P356^6.47b^ is then also exposed for hydrogen bonding with Q392^7.49b^ (Fig. 3*B*, e_4_).

In the simulations the Ramachandran plot of the backbone dihedrals of G359^6.50b^ of the *PxxG* motif clearly reveals the presence of two main clusters, associated respectively with inactive and active states (*SI Appendix*, Fig. S9*C*). Transitions between the clusters passing through ϕ=0 rad are forbidden as they would require overwinding the helix to very high-energy conformations. Conversely, the transition in the opposite direction describes the unwinding of the region by one turn and the coordinated downward shift of TM6, a hallmark of activation.

Comparison of the backbone dihedrals of the *PxxG* motif in the three states observed in our simulations and in available X-ray or cryo-EM structures of class B GPCRs highlights the crucial involvement of the backbone of G359^6.50b^ (*SI Appendix*, Fig. S9*A*). Across inactive structures the backbone dihedrals of the motif are compatible with an α-helical conformation, while a significant shift for G359^6.50b^ in active structures can be seen. The intermediate conformation observed in the simulations reflects the incomplete transition of the ϕ dihedral. In this state the conformation of the TM6 is equivalent to the one observed in the aforementioned structure of GLP-1R, which is in an intermediate activation state (Fig. 1*B*). The minor differences between the dihedral angles observed in the simulations and those in the X-ray are due to the mutations P356^6.47b^A and G359^6.50b^A introduced to stabilize the latter.

Below the central hydrogen bond is the hydrophobic network, represented by positions such as 2.53b, 3.47b, 5.54b, 6.45b, 6.46b, 7.52b, and 7.53b (Fig. 3*A*). This apolar region is also involved in the stabilization of the active state (7). As can be seen in Fig. 3 *A* and *B*, the unwinding and extension of TM6 result in a downward motion of the side chain of L358^6.49b^ of the *PxxG* motif. This positions the leucine in a similar location to L354^6.45b^ in the inactive conformation, forming apolar contacts with the surrounding residues. This conformation contributes to maintaining the unwound conformation of the *PxxG* motif.

Following the activation traveling down the receptor toward the intracellular side of the protein, the *HETx* hydrogen bond network is found (Fig. 3*A*). This system comprises H177^2.50b^, E245^3.50b^, T351^6.42b^, and Y400^7.57b^ and involves a series of hydrogen bonds that stabilize the inactive state by anchoring the intracellular portion of TM6 to the TMD (1, 27). Mutagenesis data indicate that the network is fundamental for the function of GCGR and that T351A^6.42b^ results in highly increased basal activity of glucagon receptor (30) (*SI Appendix*, Fig. S8). In active cryo-EM structures of GCGR and other class B receptors this network is consistently broken due to the repositioning of the intracellular end of TM6 (4–12).

In the simulations the detachment of T351^6.42b^ from the partners, caused by the rearrangement of the *PxxG* motif, can be observed. In the inactive state a tight interaction is found between T351^6.42b^ and E245^3.50b^, which is progressively lost in intermediate and active states (Fig. 3*B*, i_1_). The unwinding of the *PxxG* motif results in a rotation of the intracellular end of the TM6, with the side chain of the threonine residue of the *HETx* network facing away from the protein core (Fig. 3*A*). In the intermediate conformation this network is only partially disrupted. Although T351^6.42b^ is still positioned toward the core of the receptor, the distance from the partners does not allow for formation of the hydrogen bonds.

Finally one last polar network is involved in the stabilization of the inactive state and involves R173^2.54b^, R346^6.37b^, N404^8.47b^, and E406^8.49b^ (6) (intracellular hydrogen bond network, Fig. 3*A*). In particular, this network contributes to anchoring the intracellular end of TM6 to the loop between TM7 and H8, as well as H8 itself. Disruption of the network by activation of the receptor results in loss of hydrogen bonding with position 6.37b (Fig. 3*B*, i_3_), while the side chain of R173^2.45b^ is able to interact with G_α__s_, as discussed below, or take part in the *HETx* network by forming a salt bridge with E245^3.50b^ in absence of the intracellular partner (Fig. 3*B*, i_4_).

Crystal structures of GCGR and GLP-1R in complex with different allosteric antagonists show extensive contacts with the intracellular hydrogen bond network (15, 16). The antagonists intercalate a carboxyl or tetrazole group between the intracellular ends of TM6 and TM7 and form hydrophobic contacts with TM6 and TM5. These contacts might consolidate the network and thus stabilize the inactive conformation.

To test this, we run unbiased molecular dynamics simulations of GCGR in complex with glucagon or with the allosteric antagonist MK-0893 (15). We indeed observe a significant stabilization of the intracellular ends of TM6 and TM5 (*SI Appendix*, Fig. S10). Principal component analysis of the two helices shows the overall increase in rigidity of the region (*SI Appendix*, Fig. S9*B*). In particular, the carboxyl group of the compound interacts with R346^6.37b^, strengthening its interaction with E406^8.49b^ and thus preventing the extension of the intracellular end of TM6 away from the core of the receptor (*SI Appendix*, Fig. S10*C*). The absence of glucagon bound to the TMD domain of the receptor results in marked destabilization of the conformation of the NTD, which collapses against the TMD, occluding the cavity, in line with previous computational results (31) and recent cryo-EM data of GLP-1R (32) (*SI Appendix*, Fig. S9 *C* and *D*). The rearrangement of the NTD results in an increase of the tilt angle of the domain with respect to TM1, mediated by the flexibility of the stalk region, and the available volume of the extracellular TMD cavity undergoes a drop from ≈3 ${nm}^{3}$ to $\approx 1{nm}^{3}$.

### Glucagon Signaling Alone Does Not Lead to Full Activation.

In the metadynamics simulation of activation of GCGR, the state of the intracellular networks (*HETx* motif and intracellular hydrogen bond network) is partially decoupled from the rearrangement of the central hydrogen bond network and the *PxxG* motif. This is the case of the intermediate state, where loss of hydrogen bonding that stabilizes the inactive conformation in the intracellular side is observed independently of full transition of the *PxxG* motif and TM6 (Fig. 3*B*, i_1_ and i_2_ and *SI Appendix*, Fig. S11). This suggests that the conformational transition of the receptor induced by glucagon may not be sufficient for achieving full activation. Instead, combined action of the peptide and G protein is needed for the rearrangement of both extracellular and intracellular motifs and networks, via an induced fit or a mixed conformational selection/induced-fit mechanism.

This would be consistent with the fact that in cryo-EM experiments, fully active conformations of class B GPCRs have been observed only in the presence of an intracellular protein partner (4–12). Moreover, although it is generally assumed that GPCR activation is allosterically elicited by the binding of the agonist, extensive experimental and computational evidence for a number of class A GPCRs is available and indicates a similar activation process, with intracellular partners being required for the stabilization of the active state (33, 34). NMR and double electron-electron resonance experiments show the inability of extracellular agonists to fully activate $\beta_{2}$AR and A_2A_R in absence of G-protein mimetics (35, 36) and are supported by molecular dynamics simulations (37). While binding of agonists promotes preactivation, full shift of the population to the active state is dependent on interaction of G proteins or G-protein mimetics with the intracellular side of the receptor (38, 39).

To further test this hypothesis, the free-energy landscape was projected as a function of the distance between the intracellular halves of TM6 and TM3 and the ϕ dihedral angle of G359^6.50b^ by recovering the unbiased population distribution of these observables using a reweighting algorithm (40) (Fig. 1*D*). The associated reweighted free-energy surface hints at a clear path that connects inactive and active states and suggests an active role of the G protein in inducing the full activation. Starting from the inactive conformation, the TM6–TM3 distance increases from 1.4 to 2.5 nm with minor change of the ϕ value. Full activation is then observed when the dihedral angle transitions from around $\frac{3}{2}\pi$ to $\frac{\pi }{2}$ rad, unwinding the *PxxG* motif. The opposite order of events, involving hinge unwinding and then increase of the TM6–TM3 distance, is characterized by a much higher free-energy barrier (Fig. 1*D*). During the activation process the system transitions across intermediate values, represented by the intermediate state of activation. After overcoming a barrier at d(TM6,TM3) = 2.3 nm, the ϕ dihedral of the glycine residue undergoes full rearrangement. Together with TM6, the distance of TM5 from the core of the receptor also increases, in line with what is observed in active structures of GCGR and other class B GPCRs. This path, involving therefore an initial increase of the TM6–TM3 distance and then full rearrangement of G359^6.50b^ dihedrals and the high-energy penalty associated with the fully active state of the receptor (Fig. 1 *A* and *D*, state 3), supports the need of the simultaneous presence of both the agonist and the intracellular partner.

### G_α__s_ Protein Coupling Is Required for Full Activation.

The high free-energy penalty associated with the fully active receptor in the binary complex suggests an active role of G protein (induced fit) in the activation dynamics of the glucagon receptor. To verify this hypothesis we computed the free energy associated with the activation of the glucagon receptor and the coupling of G_α__s_. The free-energy landscape associated with the coupling between the two proteins was calculated using a similar setup to that of the previous simulation. Using *CV*_*Prog*_ and *CV*_*Dist*_ to sample the activation of the receptor, an additional CV was introduced to explore the binding between the receptor and G_α__s_. The CV was calculated as the *z*-axis component of the distance vector between the α5 of G_α__s_ and the intracellular side of the receptor, with the membrane extending on the *xy* plane. Y391^G^_Cα_ of the _α_5 of G_α__s_ and the geometrical center of the alpha carbon atoms of H177^2.50b^, E245^3.50b^, and Y400^7.57b^ of the *HETx* network were used for defining the vector.

**Fig. 4.**
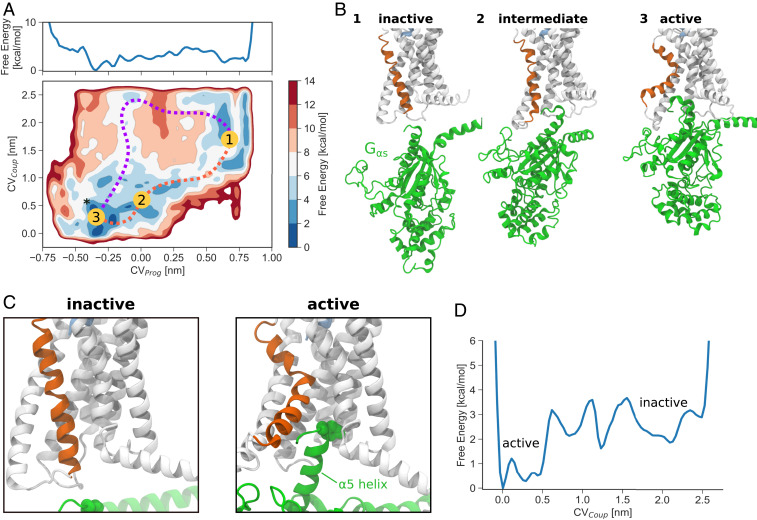
Free-energy landscape of glucagon receptor activation and G_α__s_ coupling. (*A*) Projection of the free-energy landscape onto *CV*_*Prog*_ and *CV*_*Coup*_, as well as onto *CV*_*Prog*_ alone. The asterisk indicates the CV space position of the cryo-EM structure of active GCGR. (*B*) Representative conformations of inactive, intermediate, and active states from the metadynamics simulation. (*C*) Zoomed-in view of conformations of the receptor in the inactive and active states. Y391^G^ is shown as spheres. (*D*) Projection of the free-energy landscape onto the G_α__s_ coupling coordinate, *CV*_*Coup*_. Coupled active and uncoupled inactive states can be seen.

The projection of the free-energy landscape onto *CV*_*Prog*_ and *CV*_*Coup*_ highlights a shift in the relative energy of the main states (Fig. 4*A*), with the active state being now the most favorable, in stark contrast to the binary complex (Fig. 1*A*). Starting from the inactive state (Fig. 4*B*) the G_α__s_ is still fully detached and the conformation of TM6 and TM5 corresponds to a closed intracellular cavity. The main intermediate state along the activation pathway (Fig. 4*A*, orange) is associated with loose interaction between the receptor and G_α__s_ (preassociated complex). This state is shown in Fig. 4*B*. In the intermediate state a partial opening of the intracellular cavity is observed, driven by the disruption of the *HETx* motif. Due to the limited opening of TM6 and TM5, only a shallow coupling between the two partners is observed. Finally, in the active state the rearrangement of TM6 and TM5 induces the formation of the cavity where the α5 helix of G_α__s_ can bind (Fig. 4*B*).

The landscape shows how activation of the receptor and coupling with G_α__s_ are concerted events, with the most favorable activation path involving induced fit by the protein (Fig. 4*A*). Starting from the inactive state, simultaneous conformational change of the receptor (*CV*_*Prog*_) and G_α__s_ coupling distance (*CV*_*Coup*_) drive the system to an active and coupled state. An alternative conformational selection mechanism is also possible (from 1 to 3 counterclockwise) where the cavity first opens and then G_α__s_ binds, but it is associated with much higher free energy.

**Fig. 5.**
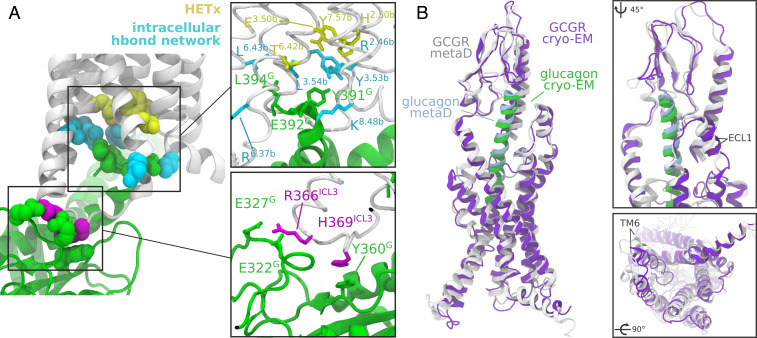
Interactions between GCGR and G_α__s_ and comparison of the active state of the receptor. (*A*) Interactions between glucagon receptor and G_α__s_ in the active state as observed in the metadynamics simulations. The *HETx* motif is colored in yellow. The boxes show the residues involved in the interaction between the C-terminal end of the α5 helix of G_α__s_ and the receptor and between G_α__s_ and ICL3. (*B*) Comparison between the active conformation of GCGR bound to G_s_ in the cryo-EM structure (4) and in the metadynamics simulation in presence of G_α__s_. The boxes show a zoomed-in view of NTDs and the intracellular side of the receptors. The structures were aligned onto the backbone atoms of their transmembrane domains.

It is possible to observe how the free energy changes during the interaction between glucagon receptor and G_α__s_ by projecting the free-energy landscape onto *CV*_*Coup*_ (Fig. 4*D*). The unbound state at *CV*_*Coup*_ >2 nm is associated with a higher energy and full solvation of G_α__s_. During the coupling process local minima, corresponding to a preactivated complex formed by the two proteins, can be observed; this is stabilized by a number of contacts, as well as stable interactions between intracellular loop 2 or 3 (ICL2 or ICL3) and G_α__s_ such as stacking between H369^ICL3^ and Y360^G^ and salt bridges between R366^ICL3^ and E322^G^ or E327^G^ (Fig. 5*A*). From this conformation, G_α__s_ can then contribute to the conformational change of TM6, resulting ultimately in fully active states.

In the active state the interaction of the C terminus of the α5 helix of G_α__s_ is in line with what is observed in cryo-EM structures of GCGR and other class B GPCRs. The key Y391^G^ side chain is hosted in a pocket defined by R173^2.46b^, Y248^3.53b^, and L249^3.54b^. E392^G^ can form salt bridges with positively charged residues such as K405^8.48b^, while L394^G^ interacts with a hydrophobic region that includes L249^3.54b^, I352^5.57^, and L352^6.43b^ (Fig. 5*A*). This set of residues of glucagon receptor is located in the proximity of the *HETx* motif, and the interaction of the receptor with G_α__s_ stabilizes the active state marked by a broken interaction between threonine T351^6.42b^ and the other members of the motif.

The polar network involving R173^2.54b^, R346^6.37b^, N404^8.47b^, and E406^8.49b^ in the intracellular portion of glucagon receptor is incompatible with G-protein binding. Indeed, in our simulation the hydrogen bonds are lost, forming instead interactions such as the stacking between R173^2.46b^ and Y391^G^ and contact between K405^8.48b^ and E378^G^. R346^6.37b^ conversely is generally fully solvated (Fig. 5*A*).

Comparison of the active state of the glucagon receptor observed in the simulations with the recently published structure of the active conformation (4) reveals a remarkable agreement of the positioning of the TMD, with significant involvement of TM6 and TM5 in both structures (Fig. 5*B*). The location of the N terminus of glucagon in the TMD binding site and the contacts of the peptide with ECL1, the stalk region, and NTD are consistent in the two models. The stability of the stalk and NTD ensures tight interaction with glucagon, and in both models the typical V shape of the TMD binding site is observed. In the intracellular side, the cavity created upon activation shows a very similar outward motion of TM6 and TM5, resulting in consistent positioning of the transmembrane helices and similar geometry of the intracellular cavity where G_α__s_ binds.

## Conclusions

The analysis of the activation dynamics of glucagon receptor and its coupling with G_α__s_ provides a detailed view of the transmission of glucagon signaling to the intracellular side of the cell membrane. The rearrangement of conserved motifs and networks enables a conformational change of GCGR that results in an intermediate state that allows for full activation after binding to the G protein.

**Fig. 6.**
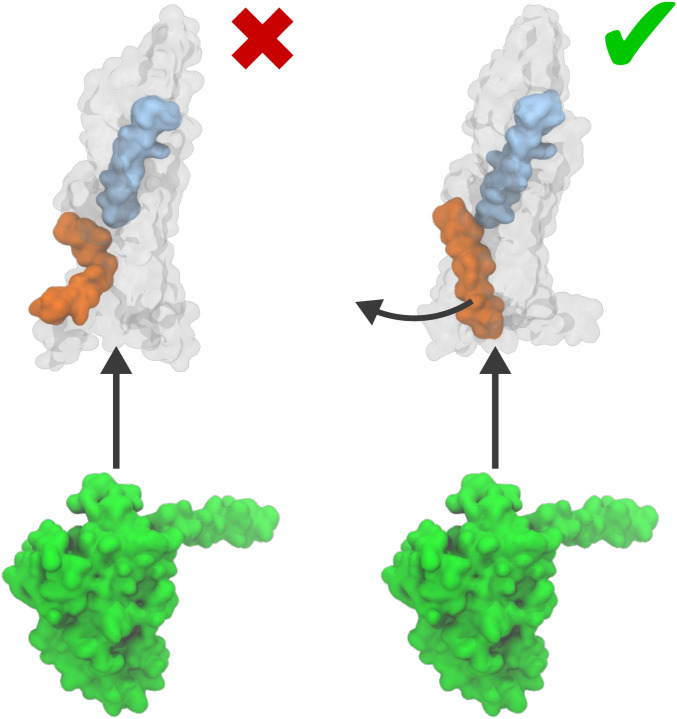
Activation models of glucagon receptor. Shown is a representation of the standard model of conformational selection in G-protein coupling (*Left*) and an induced-fit mechanism, proposed in this work (*Right*).

The computed free-energy landscapes, structural analysis, and comparison with available cryo-EM data suggest a combined mechanism for receptor activation that requires the action of both the glucagon and G protein for the full activation of the receptor (Fig. 6). The agonist first binds to the GPCR, leading to a partial activation that does not induce full rearrangement of TM6 or the complete opening of an intracellular cavity.

The most probable activation mechanism (Fig 4*A*, orange) corresponds to the G protein first forming a preassociated complex and then reaching its final position, stabilizing the active state by forming a number of polar and hydrophobic contacts with the intracellular cavity. A second mechanism where the receptor is first activated by the extracellular agonist is associated with much higher free energy (Fig 4*A*, purple). Thus, both induced-fit and conformational selection mechanisms are possible, but the former is more favorable. Multiple mutagenesis studies of the residues that, according to our model, play a pivotal role in the activation mechanism confirm their biological importance (4, 41–51).

Our work reveals an active role of the G protein in the activation process and complements the experimental findings on the glucagon receptor and other class B GPCRs with information about the conformational dynamics of these crucial processes. Analysis of the simulations shows remarkable agreement of inactive and active states with X-ray and cryo-EM data and provides a structural model of an intermediate state. This study offers a rationale for the mode of action of allosteric antagonists of the glucagon receptor that lock TM6. It explains the stabilization of the helix induced by these compounds and their effect in consolidating polar networks that impede TM6 rearrangement, thus preventing the full coupling of the G protein and the activation of the receptor.

## Materials and Methods

### System Setup.

The X-ray structure of glucagon receptor [PDB 5YQZ (26)] bound to a glucagon analogue was used for the metadynamics simulations. The fused T4 lysozyme was removed and mutations reverted to wild type using MODELLER (52). The glucagon analogue was mutated to glucagon. For the simulation of receptor activation and G-protein coupling, human G_α__s_ [PDB 6EG8 (53)] was used, reverting mutations to the wild-type amino acids. The systems were embedded in a preequilibrated dioleoylphosphatidylcholine membrane patch (54) and solvated in TIP3P water (55), charges were balanced with chloride ions, and for the ternary complex a concentration of 150 mM NaCl was used. The systems were then parameterized using AMBER14SB (56) and LipidBook (54) parameters.

### Molecular Dynamics and Metadynamics Setup.

Molecular dynamics and metadynamics simulations were performed using GROMACS 2016.3 (57) and PLUMED 2.4.3 (58).

After equilibration, the activation free energy of glucagon receptor in absence of G_α__s_ was computed using parallel tempering well-tempered metadynamics (19) in the well-tempered ensemble (59), using 12 replicas covering the 300- to 360-K temperature range. As presented in Results and Disc*ussion*, a set of two CVs was used: *CV*_*Prog*_ is the difference between the RMSD_Cα_ of TM6 of the starting inactive structure and that of the cryo-EM structure of active GLP-1R [PDB 5VAI (27)], and *CV*_*Dist*_ is the sum of the two values.

For the calculation of the free-energy landscape of glucagon receptor activation and G_α__s_ protein coupling, the *CV*_*Coup*_ CV was defined as the distance between Y391^G^_Cα_ and the center of the alpha carbon atoms of H177^2.50b^, E245^3.50b^, and Y400^7.57b^ of the *HETx* motif. The well-tempered metadynamics simulation was run in the multiple-walkers (60) scheme using 12 walkers at 300 K.

In both sets of simulations hills were deposited every 500 integration steps, with an initial height of 1.5 kJ/mol and a bias factor of 15. The Gaussian sigma was set to 0.05 nm for all CVs. The metadynamics simulations were terminated when thorough exploration of the relevant CV space was achieved, and the estimates of activation free energy adopted an asymptotic behavior. A total of 4.0 μs of aggregate sampling was performed for the simulation of GCGR in absence of G_α__s_, while 12.7 μs accumulated for the simulation in presence of G_α__s_. All metadynamics production runs were performed in the canonical ensemble (61).

Unbiased molecular dynamics of glucagon receptor in complex with glucagon or with the allosteric antagonist MK-0893 were performed. One single 1-μs-long trajectory was computed for each system, in the isothermal–isobaric ensemble at 300 K and 1 bar. MK-0893 was parameterized with GAFF2 (62) and AM1-BCC charges (63).

MDTraj (64), VMD (65), and PyMol (Schrödinger) were used for data analysis and visualization.

### Data Availability.

Metadynamics input files can be found on PLUMED NEST (https://www.plumed-nest.org/): plumID:20.006. Models, topologies, molecular dynamics input files, and other relevant data are available on GitHub: https://github.com/Gervasiolab/Gervasio-Protein-Dynamics/raw/master/GCGR-metad/NEST.zip.

## Supplementary Material

Supplementary File
