# Supplementary material for: A combined activation mechanism for the glucagon receptor
Source: Proc Natl Acad Sci U S A. 2020 Jun 22;117(27):15414–22. doi: 10.1073/pnas.1921851117 (PMC7355025; doi:10.1073/pnas.1921851117)
Supplement: Supplementary File [file pnas.1921851117.sapp.pdf]

PNAS
www.pnas.org

Supplementary Information for

A Combined Activation Mechanism for the Glucagon Receptor

Giulio Mattedi, Silvia Acosta-Gutiérrez, Timothy Clark, Francesco L. Gervasio

Francesco L. Gervasio
E-mail: f.l.gervasio@ucl.ac.uk

This PDF file includes:

Supplementary text
Figs. S1 to S13
References for SI reference citations

www.pnas.org/cgi/doi/10.1073/pnas.1921851117

Supporting Information Text

Simulation Setup

After building the systems, energy minimization was performed using a steepest descent algorithm and stopped when the maximum force exerted on any atom dropped below 1000.0 kJ/mol/nm. An equilibration procedure followed: in the NPT ensemble the systems were progressively heated to 300 K over 60 ns while applying a restraint on alpha carbon atoms of the proteins. In the last 20 ns the restraint were released.

For the parallel tempering well-tempered metadynamics (1) simulation in the well-tempered ensemble (2, 3) of glucagon receptor activation in absence of $G_{\alpha s}$, 12 replicas of the system were further equilibrated to the 300-360 K range in the NPT ensemble. The temperature distribution was determined with the geometric progression:

$$T_i = T_0 \cdot e^{ki} \quad [1]$$

where T_i is the temperature of the i-th replica (0-indexed), $T_0 = 300$ is the lowest temperature, and $k = 0.016575$ was selected to cover the target temperature range with 12 replicas.

After equilibration of this system, a 10 ns-long parallel tempering well-tempered metadynamics simulation was performed using the potential energy of the system as collective variable in the NVT ensemble. Hills were deposited every 500 steps, with an initial height of 5 kJ/mol, a sigma of 1000 kJ/mol and a bias factor of 20 (Fig. S1). Following this preparatory metadynamics run, the production runs were performed by biasing CV_{Prog} and CV_{Dist} as described in the main text and in the section below. The bias deposited on the potential energy space was loaded during these runs. In both simulations exchanges between replicas were attempted every 1000 steps.

For the simulations of glucagon receptor activation and $G_{\alpha s}$ the system was minimized and equilibrated to 300 K with the same setup, without bias on the potential energy. 12 walkers were used for the multiple walkers (4) well-tempered metadynamics simulation, starting in different positions along the activation path.

In all molecular dynamics simulations a timestep of 2 fs was used. Temperature was enforced with the v-rescale thermostat (5) and, in NPT simulations, the Parrinello-Rahman barostat was employed (6). Electrostatics were treated with the PME-Switch algorithm, and the nonbonded interaction cutoff was set to 1 nm.

Collective Variables. The CV_{Prog} and CV_{Dist} collective variables were used for enhancing the activation of glucagon receptor. The $RMSD_{C\alpha}$ of TM6 ($K334^{6.35}$ to $E371^{ECL3}$ in GCGR) was calculated with respect to the conformation adopted in the starting inactive conformation of glucagon receptor (PDB 5YQZ (7)) and the active glucagon-like peptide 1 receptor (GLP-1R, PDB 5VAI (8)). The active conformation of GLP-1R was used as, when the simulations were performed, active X-ray/cryo-EM structures of GCGR were not available. The $RMSD_{C\alpha}$ to the two reference structures was measured after alignment of the systems onto a set of alpha carbon atoms of stable residues of the TMD, excluding TM6. CV_{Prog} was then defined as the difference between $RMSD_{C\alpha}^{active}$ and $RMSD_{C\alpha}^{inactive}$, while CV_{Dist} was the sum of the two values. The exploration of the CV_{Dist} CV was limited to values below 1.6 nm with a repulsive potential.

Following the simulations, the activation free energy landscape of GCGR in absence of $G_{\alpha s}$ was projected as function of the distance between the intracellular ends of TM6 and TM3, and the ϕ dihedral angle of $G359^{6.50b}$ by reweighting (9). The TM6-TM3 distance was monitored using the geometric center of the alpha carbon atoms of $G246^{3.51}$, $L247^{3.52}$, $Y248^{3.53}$, $L249^{3.54}$, $H250^{3.55}$, and $Y343^{6.34}$, $K344^{6.34}$, $F345^{6.34}$, $R346^{6.34}$, $L347^{6.34}$.

In the simulation of activation of GCGR and coupling of $G_{\alpha s}$, the interaction of glucagon receptor with G protein was biased with the CV_{Coup} collective variable, defined as the Z-axis projection of the distance vector between $Y391^{G}{}_{C\alpha}$ and the center of the alpha carbon atoms of $H177^{2.50b}$, $E245^{3.50b}$ and $Y400^{7.57b}$. A harmonic repulsive potential discouraged the exploration of the CV above 2.5 nm. The orthogonal exploration of $G_{\alpha s}$ was limited to the relevant space by applying a similar potential on the XY-plane components of the same vector, that was triggered at values above 1.5 nm. Furthermore, following the example of Saleh et al. (10), the myristoylation of the N-terminus of $G_{\alpha s}$ was modeled as an harmonic restraint on the distance between the protein and the inner leaflet of the membrane.

Unbiased Molecular Dynamics. Molecular dynamics simulations of glucagon receptor in complex with glucagon or with the allosteric antagonist MK-0839 were run. The ligand was parametrized using AM1-BCC charges (11) and GAFF2 parameters (12). The systems were equilibrated to 300 K and 1 bar with the procedure described above. For the system including MK-0839 the equilibration included positional restraints of the atoms of the compound, and an ion concentration of 150 mM NaCl was used. One single 1 μs-long unbiased MD trajectory in the NPT ensemble was accumulated for each system.

Interactions of Glucagon with the NTD and ECL1

During the parallel tempering metadynamics simulations of glucagon receptor activation, glucagon remained stably bound to the receptor. Although the stalk region ($G125$-$K136^{1.34b}$) that connects the NTD with the TMD shows a degree of flexibility, the NTD maintained its conformation and stabilized the C-terminal portion of the peptide throughout the trajectory. Three disulfide bonds are responsible for maintaining a well structured fold of the region, namely $C43^{NTD}$-$C67^{NTD}$, $C58^{NTD}$-$C100^{NTD}$ and $C81^{NTD}$-$C121^{NTD}$. These, together with the connection of the domain with the stalk region and the contacts with ECL1, result is a stable positioning of the NTD with respect to the TMD.

The interaction of glucagon with the receptor is favored by a large number of contacts with the TM binding site, TM1, ECL1, the stalk and the NTD. A set of apolar interactions involve residues such as F6^{P}, L14^{P}, F22^{P}, V23^{P}, and L26^{P}. Polar interactions are also of great importance, and contribute to the stability of the complex. Tight polar contacts highlighted in our simulations are Q131$^{1.29b}$-R17^{P}, W215^{ECL1}-R18^{P}, R385$^{7.35b}$-D9^{P}, and D385$^{7.42b}$-S2^{P} (Fig. 2 of the main text).

Extra cellular loop 1 (ECL1) of Class B GPCRs is especially prominent, and plays a key role in aiding ligand binding to the extracellular vestibule. In a number of X-ray structures of glucagon receptor without agonist peptides this loop is either missing (13, 14) or locked in a β-hairpin conformation by the antigen-binding fragment of an inhibitory antibody cocrystallised with the scope of stabilizing the extracellular region. In the initial conformation of the system, as well as peptide-bound structures of GLP-1R (8, 15, 16), the region adopts an α-helical geometry. In our simulations extensive contacts between the loop and R18^{P}, F22^{P} and W25^{P} stabilize its conformation and aid the recognition of the peptide by glucagon receptor. In the active structure of GLP-1R the longer loop presents rather a bent helix throughout its length, and the lack of a glycine residue at the end of the element (G219^{ECL1} in GCGR) contributes to the absence of unstructured portions. Nonetheless, the helical conformation of ECL1 of glucagon receptor observed in the starting secondary structure comprising three helical turns is maintained, in our simulations, both in inactive and active states.

Comparison between glucagon and NNC1702

In the starting X-ray structure of glucagon receptor (5YQZ (7)), the receptor is bound to the partial agonist NNC1702. The peptide, des-H1-[E9, K24(4xγE), L27]glucagon, was engineered with the aim of reducing potency while maintaining strong affinity for GCGR, and be stable and soluble at the experimental conditions (7). In the molecular dynamics and metadynamics simulations of this work, NNC1702 was mutated to wild-type glucagon (Fig. S2**a**,**b**).

Analysis of the interaction of the peptides and GCGR in the metadynamics simulations and in the X-ray structure highlights marked differences in the N-terminus of the agonists. In the simulations of GCGR bound to glucagon, H1^{P}, deleted in NNC1702, interacts with polar side chains of the TMD. In particular, the N-terminal is in proximity of E382$^{6.53b}$ and forms contact with it, which can in turn interact with Y238$^{3.44b}$ as also presented in the main text (Fig. S2**c**). This allows the peptide to directly influence the *central hydrogen bond network*, that is in direct contact with the *PxxG motif*, promoting the activation of the protein. The side chain is preferentially positioned in a pocket formed by TM3, TM5, TM6 and ECL2, in agreement with the cryo-EM structure of active GCGR (17), and is in proximity of positions such as Q232$^{3.77}$, N298^{ECL2}, W304$^{5.36}$, L307$^{5.39}$, V311$^{5.43}$. The more subtle mutation D9E^{P} could instead exert its effect via a less favorable interaction with R378$^{7.35b}$ when the residue of the peptide is a glutamate, although from our simulations the contact does not appear to directly influence the activation event.

Mutation of positions 24 and 27 were introduced to NNC1702 in order to improve stability and solubility. Indeed, by comparing the metadynamics simulations of glucagon bound to GCGR and the X-ray structure of NNC1702 bound to the receptor, only minor differences in the interaction between the peptides and GCGR can be identified. Position 24 (Q24K(4xγE)^{P}) is exposed to the solvent and the 4xγE portion, while not modeled in the crystal structure, likely interacts with water. In position 27 (M27L^{P}), both methionine and leucine provide a hydrophobic surface for interaction with Y65^{NTD}, A118^{NTD} and Q122^{NTD}, with minor differences between the two (Fig. S2**d**).

Electrostatic Potential and Ion Densities

Fig. S3, S4 show the electrostatic potential at the molecular surface of both glucagon receptor and $G_{\alpha s}$. In the case of the glucagon receptor the electrostatic potential is predominantly unfavorable for cations (+10 kT/e) in the solvent exposed regions and neutral/hydrophobic in the membrane region. The core of the TMD of the receptor (Fig. S4**c**) is predominantly positive, with the exception of E245$^{3.50b}$. $G_{\alpha s}$ (Fig S4) is mainly exposed to the solvent, and shows different patches at positive and negative values of the electrostatic potential, with the α5 helix region not being highly charged but with complementarity to the binding surface of the receptor (Fig. S4).

In order to assess the influence of the ionic environment on the interaction between glucagon receptor and the G protein, a new metadynamics simulation of the activation of glucagon receptor and $G_{\alpha s}$ coupling was performed. In the simulation a concentration of NaCl of 150 mM was used, and the bias deposited in the previous simulation in the space of the three CVs was loaded. The same multiple walkers metadynamics setup as the previous simulation was used. The 12 walkers were left to diffuse for a further 1.2 μs total sampling, and the free energy landscape was reconstructed, and is shown in Fig. 4 of the main text. A comparison of the previous free energy landscape and the one obtained with the additional sampling at 150 mM NaCl is presented in Fig. S5**a**. The landscapes indicate minimal change of the free energy surface upon addition of the ion concentration, reflecting a very limited influence of sodium and chloride in the coupling between GCGR and $G_{\alpha s}$. As can be observed in Fig. S5**b**, no specific interaction between the ions and the proteins was identified to influence the activation process. Fig. S6 shows the probability distribution function of both ion species, normalized to the bulk density, along the axis perpendicular to the membrane for the 12 walkers of the system. No hotspots were identified at the GCGR-G protein binding interface, except for transient interactions of chloride with T172$^{2.45b}$ and R52^{G} in the intermediate ensemble (Fig. S6**c**).

We then repeated the same analysis on the unbiased MD trajectory of glucagon receptor in complex with MK-0839 and in absence of glucagon at 150 mM NaCl (Fig. S7). Fig. S7**a**,**b** shows the 3D density maps for chloride and sodium; hotspots are located in the solvent accessible area around the loops, but no hotspot was identified in the TMD of the receptor. Moreover, in absence of glucagon, the collapse of the NTD reduces the access of ions to the TMD. From Fig. S7**c**, we observe that not even water is able penetrate through the TMD during the molecular dynamics run.

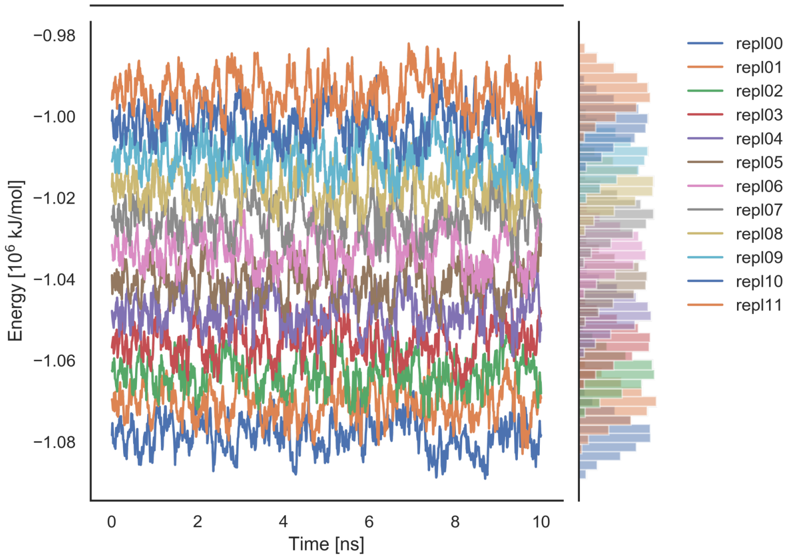

Fig. S1. Energy distribution of the parallel tempering replicas over the short metadynamics run biasing the energy, for glucagon receptor in absence of $G_{\alpha s}$.

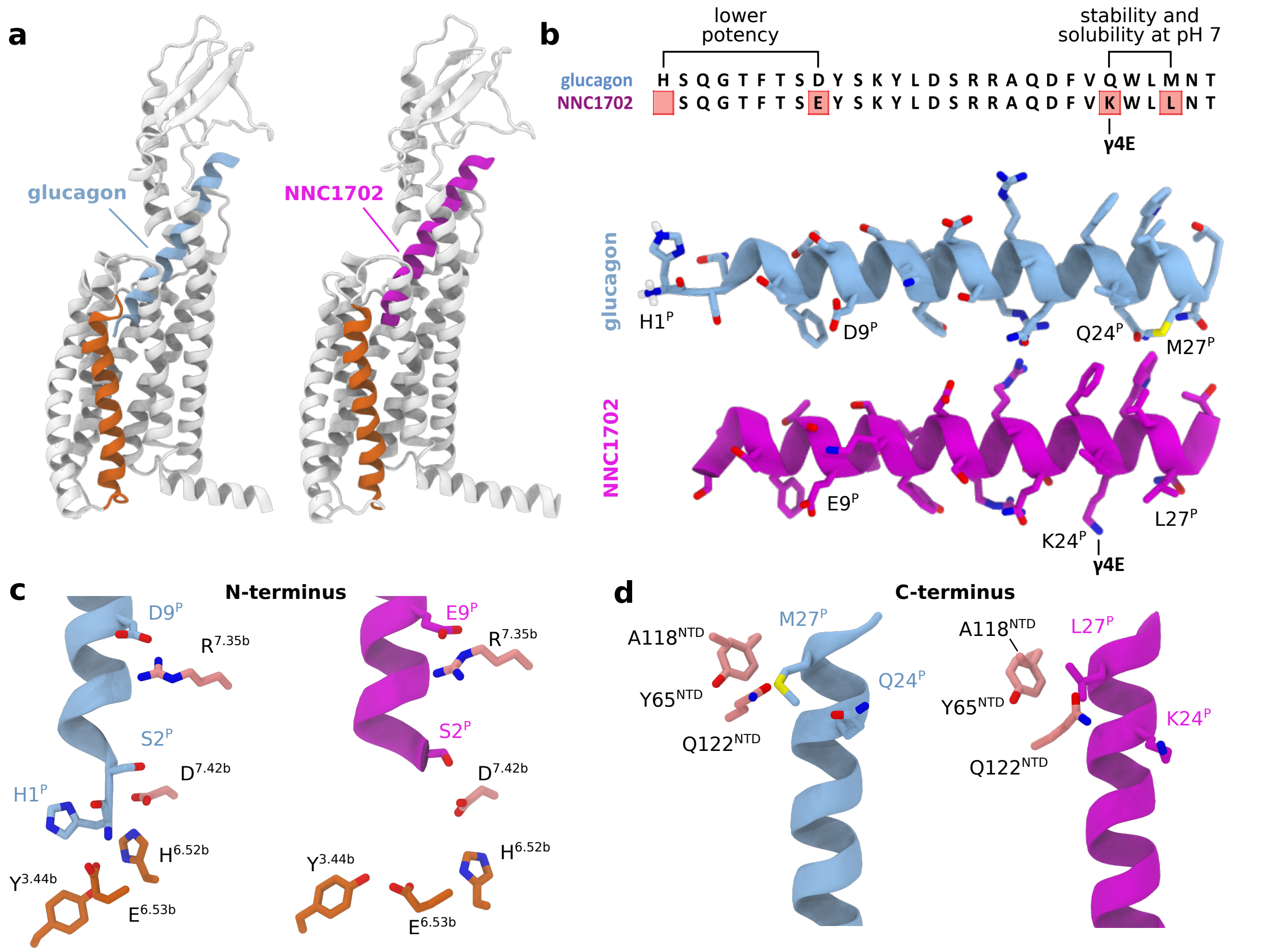

Fig. S2. Differences in the interactions of glucagon with glucagon receptor in the metadynamics simulations and of NNC1702 with the receptor in the starting X-ray structure. **a** Overview of the complexes of the receptor with the peptides. **b** Alignment of the sequences of the peptides and effect of the four mutations according to Zhang et al. (7) **c** Interactions of the N-terminal region of the peptides. **d** Interactions of the C-terminal region of the peptides.

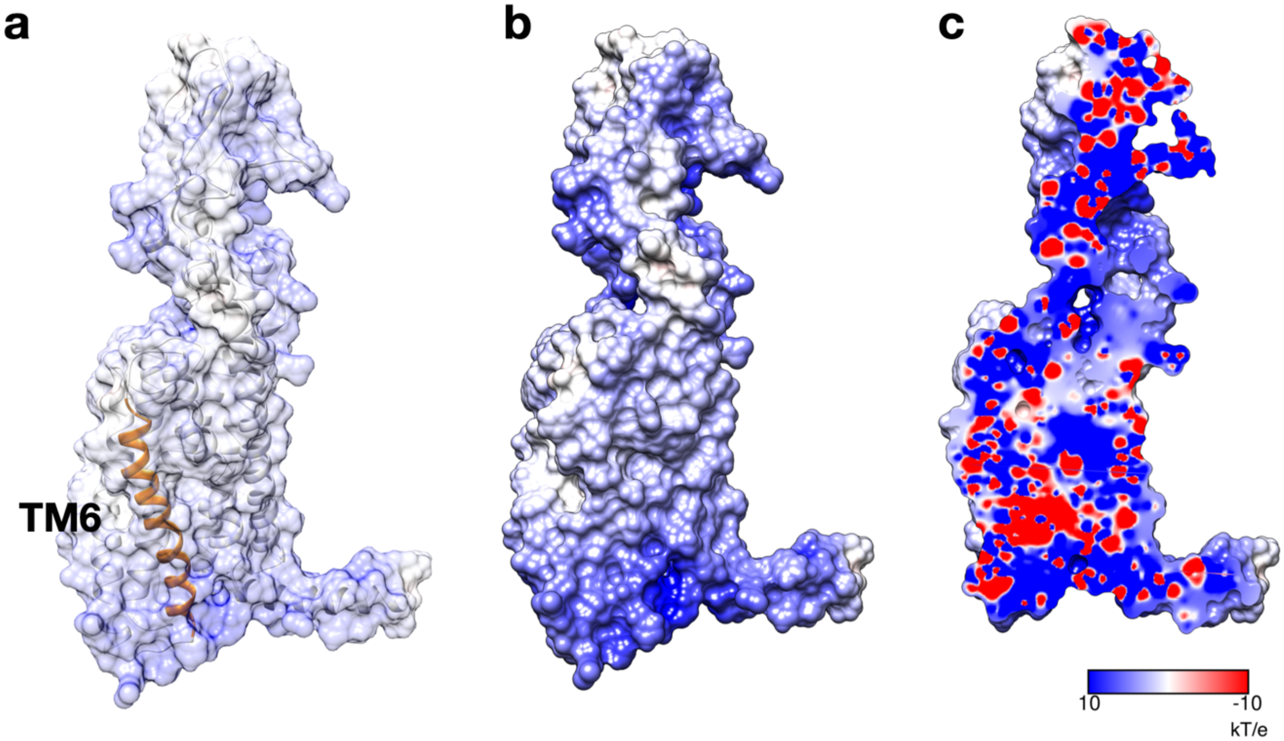

Fig. S3. Electrostatic potential at the molecular surface of the of the glucagon receptor, ranging from blue (+10 kT/e) to red (-10 kT/e). **a** Molecular surface superimposed on top of a cartoon representation of the receptor, TM6 is highlighted in orange. **b** Surface representation. **c** Longitudinal section of the TMD, showing electrostatic potential at the core of the receptor.

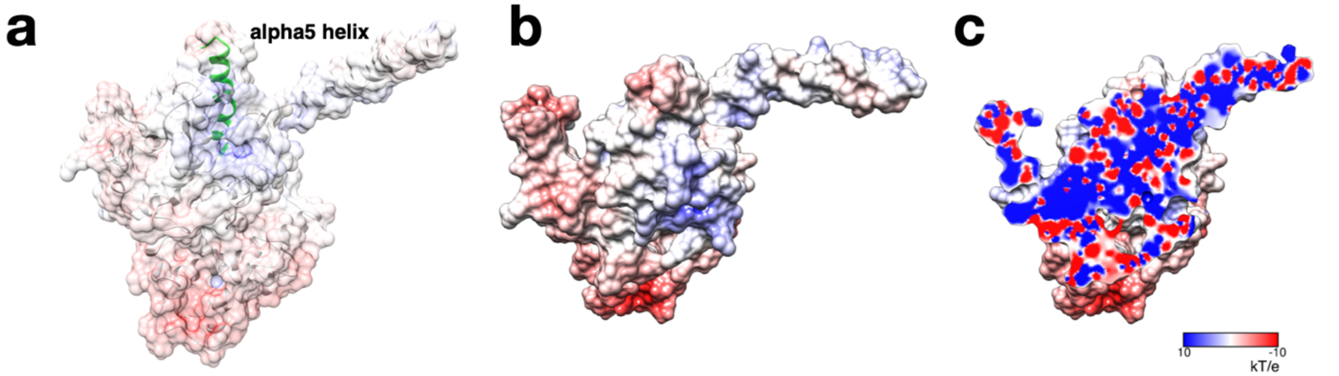

Fig. S4. Electrostatic potential at the molecular surface of the $G_{\alpha s}$, ranging from blue (+10 kT/e) to red (-10 kT/e). A top view of the protein is presented. **a** Molecular surface superimposed on top of a cartoon representation of the $G_{\alpha s}$, α5 helix is highlighted in green. **b** Surface representation. **c** Longitudinal section of the $G_{\alpha s}$.

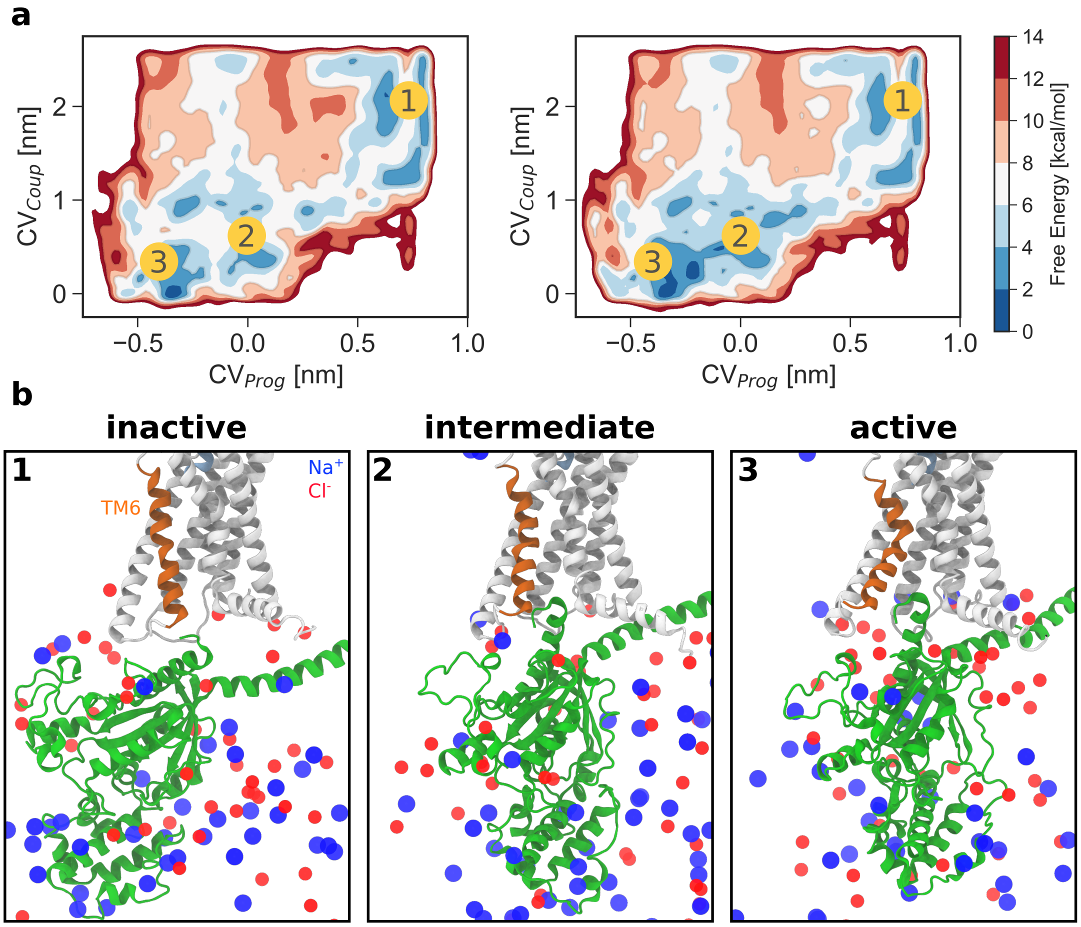

Fig. S5. Free energy landscape of GCGR activation and G protein coupling as a function of CV_{Prog} and CV_{Coup} in absence or presence of 150 mM NaCl. **a** (left) Free energy landscape in absence of salt concentration. (right) Free energy landscape after the sampling at 150 mM NaCl. **b** Representative snapshots of inactive, intermediate and active states from the sampling at the ion concentration.

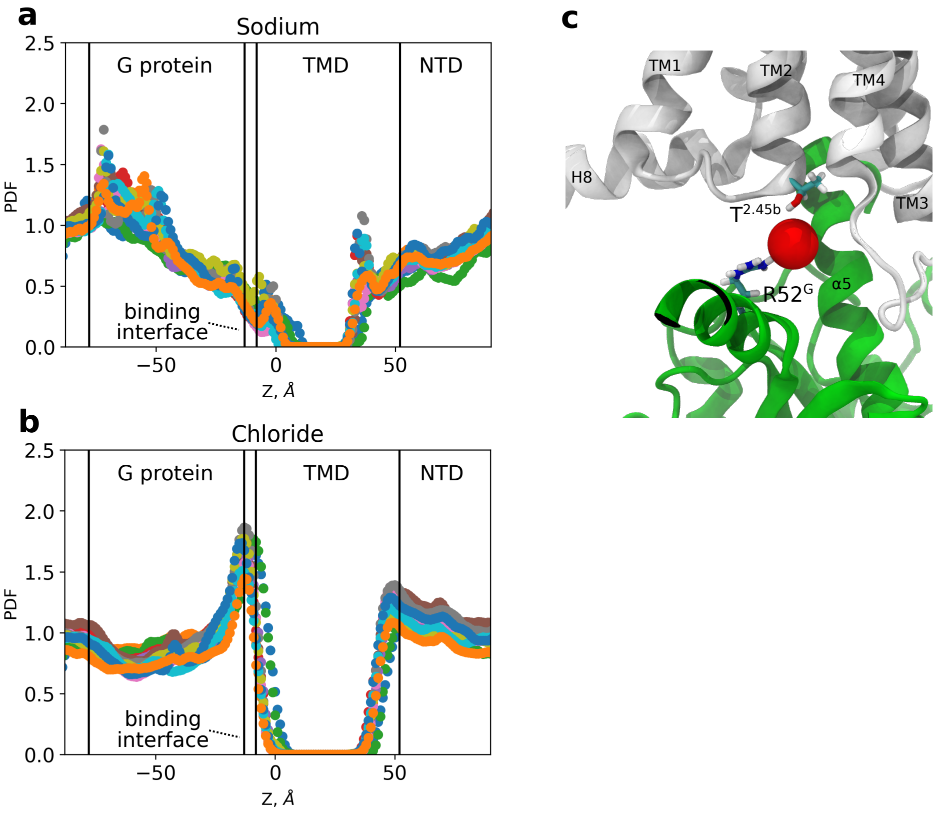

Fig. S6. Normalized probability density function (PDF) along the Z-axis, perpendicular to the membrane, for sodium and chloride ions, over the multiple walkers metadynamics of GCGR activation in presence of $G_{\alpha s}$ at 150 mM NaCl. The density for each of the 12 walkers is shown. **a** Sodium ions and **b** chloride ions. The main elements of the systems are labeled and the $G_{\alpha s}$-GCGR binding interface region is also highlighted. **c** Interaction of chloride (red vdW sphere) with T172$^{2.45b}$ and R52^{G} in the intermediate ensemble.

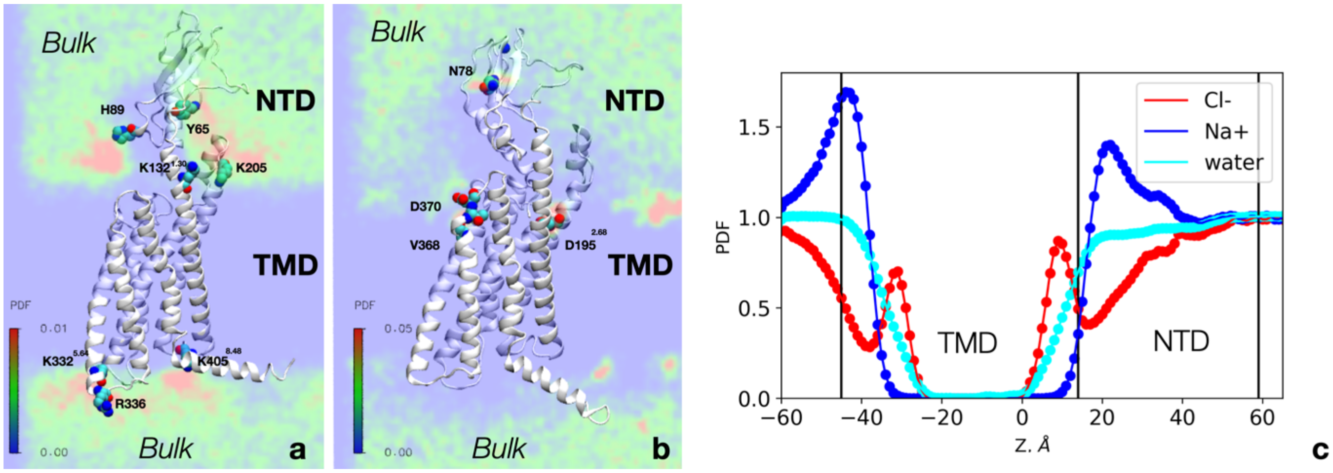

Fig. S7. Ion hotspots at 150 mM NaCl for the unbiased MD simulation of GCGR in complex with MK-0839. 3D density distribution maps are superimposed onto a cartoon representation of the glucagon receptor. Residues in a 0.3 nm radius from the ion hotspots are highlighted as spheres. **a** 3D chloride density distribution. **b** 3D sodium density distribution. **c** Normalized probability density function (PDF) along the Z-axis, perpendicular to the membrane: sodium ions (blue), chloride ions (red) and water (cyan). The main elements of the system are labeled.

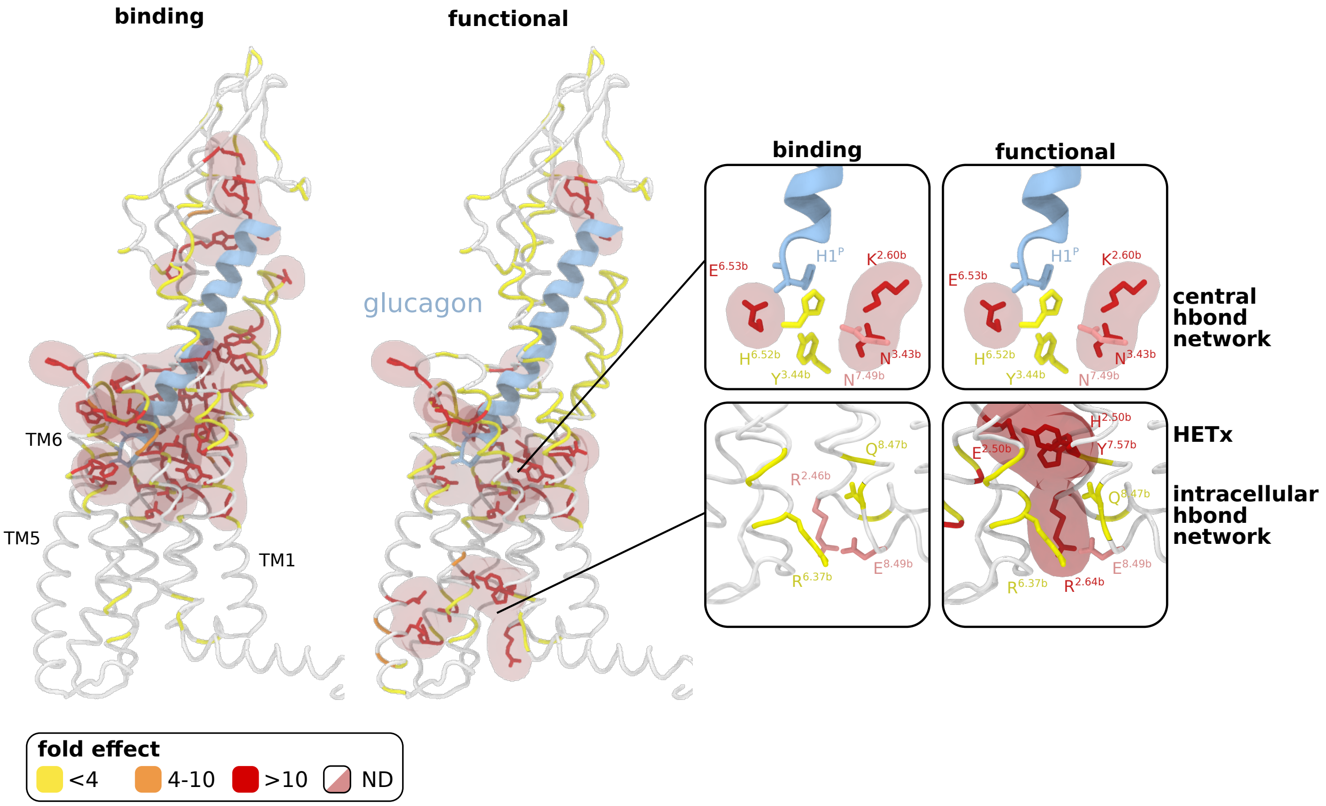

Fig. S8. Effect of mutations of the glucagon receptor on binding affinity and functional potency of glucagon (13, 17–27), overlaid on a representative conformation of inactive GCGR bound to glucagon in the metadynamics simulations. Data expressed as fold increase over wild-type.

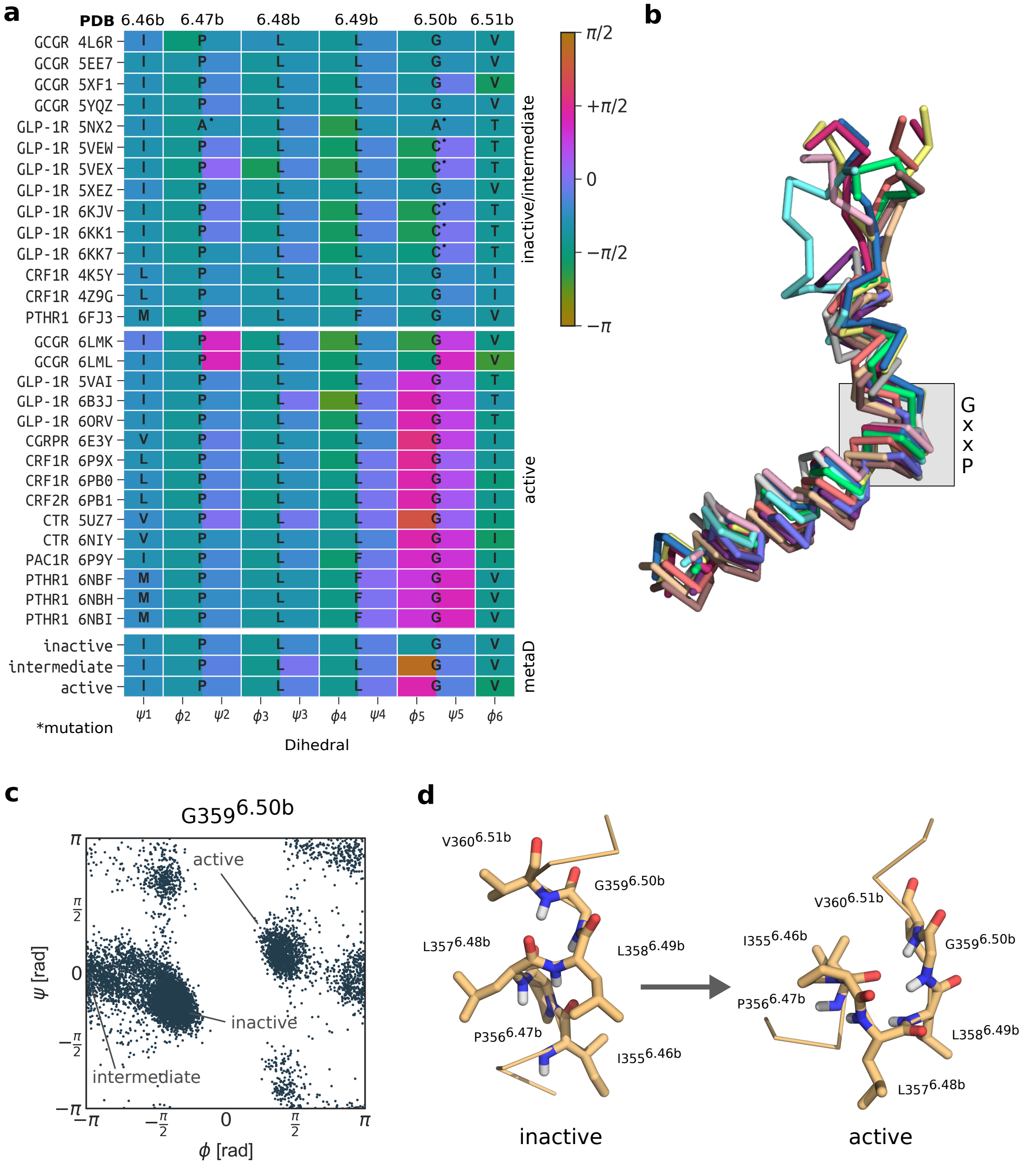

Fig. S9. *PxxG motif* backbone dihedrals across experimental structures and in the metadynamics simulations. **a** Comparison of backbone dihedrals of the *PxxG motif* of the available X-ray and cryo-EM structures of Class B GPCRs (7, 8, 13–16, 28–39). **b** Superposition of TM6 of the available active structures. **c** Ramachandran plot of G359$^{6.50b}$ over the metadynamics simulation of the glucagon receptor. Inactive, intermediate and active states can be clearly identified. **d** Representative conformations of the *PxxG motif* in the inactive and active states.

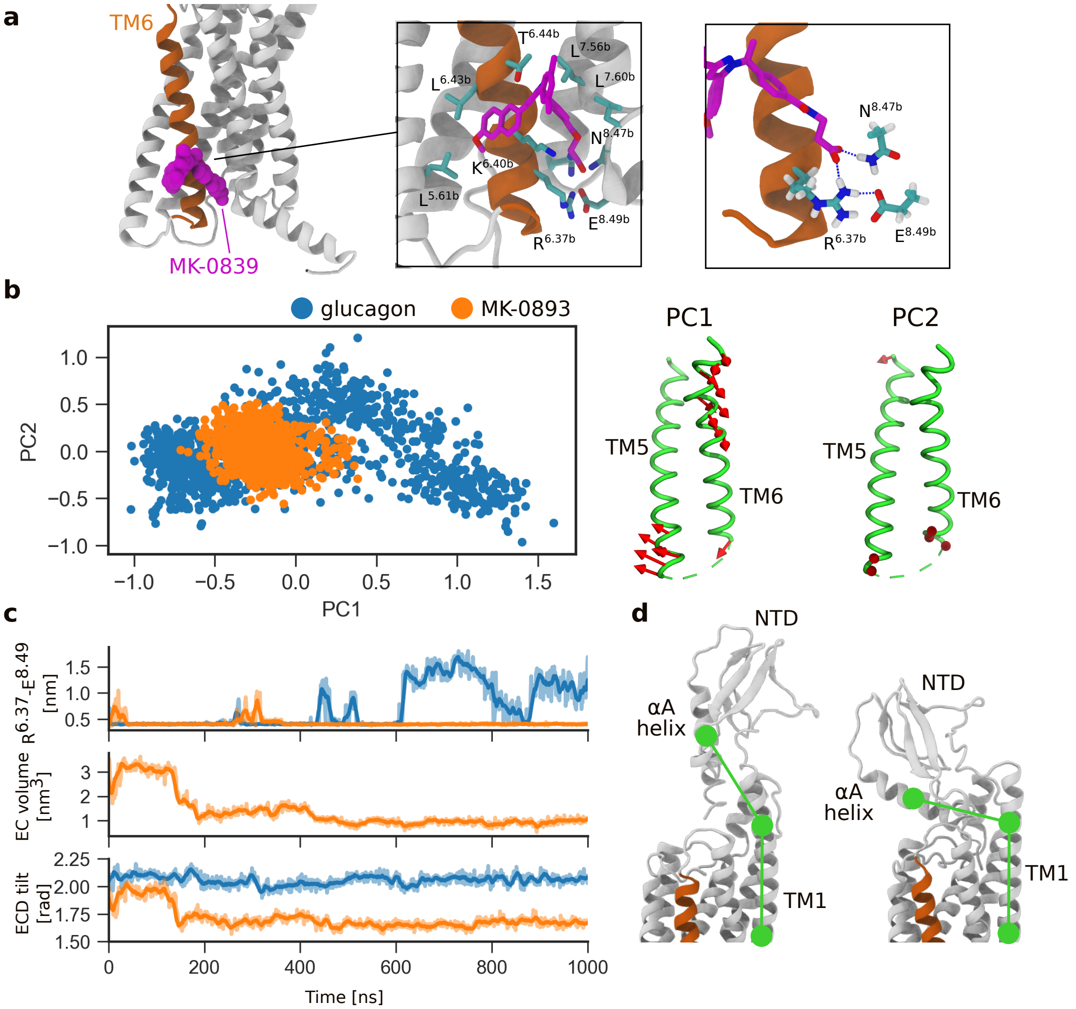

Fig. S10. Unbiased MD simulations of the glucagon receptor in complex with the allosteric antagonists MK-0893 (14) or in the apo form. **a** Interactions of the ligand with the receptor. The ligand stabilizes the intracellular ends of TM6 and TM5. **b** Principal component analysis of the α carbon atoms of TM6 and TM5 in the unbiased MD simulations of GCGR in complex with glucagon or MK-0839, after alignment onto the TMD of the receptor. **c** Distance between $R^{6.37}$ and $E^{8.49}$ of the *intracellular hydrogen bond network* over the two MD simulations. Volume of the extracellular TMD cavity in the simulation of glucagon receptor bound to the antagonist. Tilt of NTD in the two MD simulations, as calculated by the angle between the center of TM1, the beginning of TM1, and the center of the αA helix of NTD.

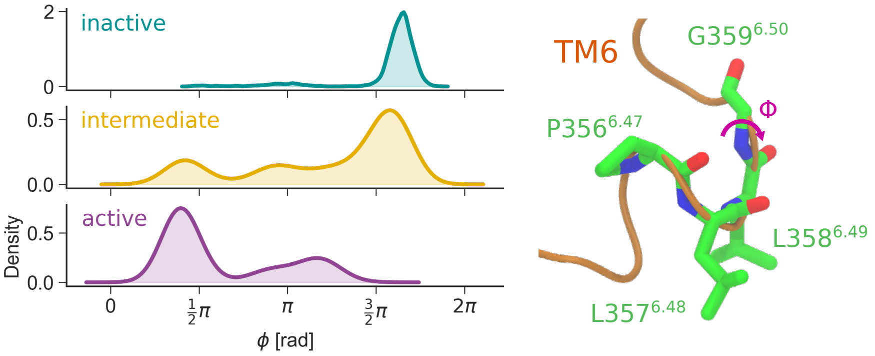

Fig. S11. Distribution of the ϕ backbone dihedral of G359$^{3.50b}$ in inactive, intermediate and active states over the metadynamics simulation of activation of GCGR in absence of $G_{\alpha s}$.

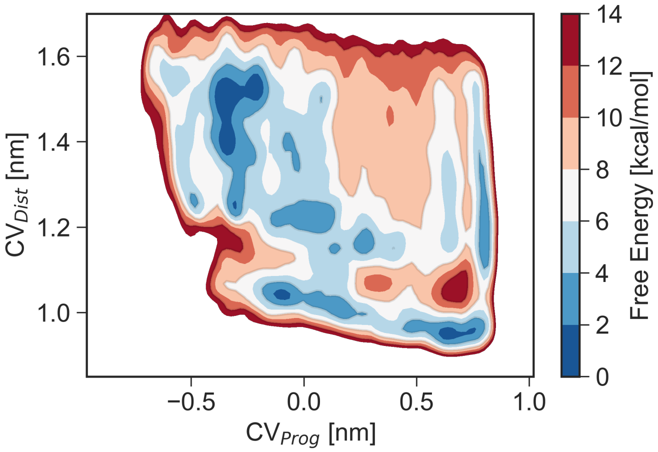

Fig. S12. Activation free energy landscape of glucagon receptor, in presence of $G_{\alpha s}$, as a function of CV_{Prog} and CV_{Dist}, at 150 mM NaCl.

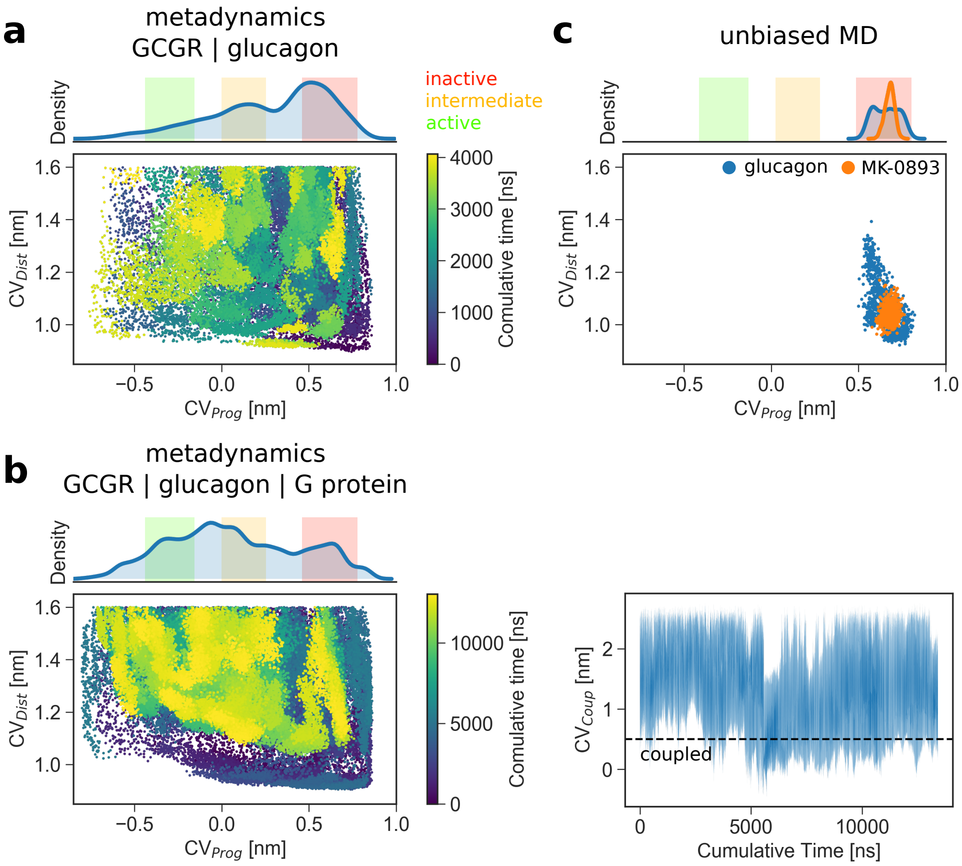

Fig. S13. CV space sampling of the metadynamics and unbiased MD simulations. The marginal plots show the probability density of the sampling of the simulations as a function of CV_{Prog}. **a** Sampling of the parallel tempering metadynamics simulation of the activation of GCGR bound to glucagon, in absence of G protein. **b** Sampling of the multiple walkers metadynamics simulation of the activation and G protein coupling of GCGR bound to glucagon, in presence of G protein. The time series of G protein coupling, CV_{Coup} is shown. **c** Projection of the unbiased MD simulations of GCGR bound to either glucagon of MK-0839 on CV_{Prog} and CV_{Dist}.

References

1. Bussi G, Gervasio FL, Laio A, Parrinello M (2006) Free-energy landscape for beta hairpin folding from combined parallel tempering and metadynamics. *J. Am. Chem. Soc.* 128(41):13435–13441.
2. Bonomi M, Parrinello M (2010) Enhanced sampling in the well-tempered ensemble. *Phys. Rev. Lett.* 104(19):1–4.
3. Deighan M, Bonomi M, Pfaendtner J (2012) Efficient simulation of explicitly solvated proteins in the well-tempered ensemble. *J. Chem. Theory Comput.* 8(7):2189–2192.
4. Raiteri P, Laio A, Gervasio FL, Micheletti C, Parrinello M (2006) Efficient reconstruction of complex free energy landscapes by multiple walkers metadynamics. *J. Phys. Chem. B* 110(8):3533–3539.
5. Bussi G, Donadio D, Parrinello M (2007) Canonical sampling through velocity rescaling. *J. Chem. Phys.* 126(1):014101.
6. Parrinello M, Rahman A (1981) Polymorphic transitions in single crystals: A new molecular dynamics method. *J. Appl. Phys.* 52(12):7182–7190.
7. Zhang H, et al. (2018) Structure of the glucagon receptor in complex with a glucagon analogue. *Nature* 553(7686):106–110.
8. Zhang H, et al. (2017) Structure of the full-length glucagon class B G-protein-coupled receptor. *Nature* 546(7657):259–264.
9. Tiwary P, Parrinello M (2015) A time-independent free energy estimator for metadynamics. *J. Phys. Chem. B* 119(3):736–742.
10. Saleh N, Saladino G, Gervasio FL, Clark T (2017) Investigating allosteric effects on the functional dynamics of β2-adrenergic ternary complexes with enhanced-sampling simulations. *Chem. Sci.* 00(Md):1–8.
11. Jakalian A, Jack DB, Bayly CI (2002) Fast, efficient generation of high-quality atomic charges. AM1-BCC model: II. Parameterization and validation. *J. Comput. Chem.* 23(16):1623–1641.
12. Wang J, Wolf RM, Caldwell JW, Kollman Pa, Case Da (2004) Development and testing of a general amber force field. *J. Comput. Chem.* 25(9):1157–1174.
13. Siu FY, et al. (2013) Structure of the human glucagon class B G-protein-coupled receptor. *Nature* 499(7459):444–449.
14. Jazayeri A, et al. (2016) Extra-helical binding site of a glucagon receptor antagonist. *Nature* 533(7602):274–277.
15. Jazayeri A, et al. (2017) Crystal structure of the GLP-1 receptor bound to a peptide agonist. *Nature* 546(7657):254–258.
16. Liang YLL, et al. (2018) Phase-plate cryo-EM structure of a biased agonistbound human GLP-1 receptor-Gs complex. *Nature* 555(7694):121–125.
17. Qiao A, et al. (2020) Structural basis of Gs and Gi recognition by the human glucagon receptor. *Science (80-.).* 367(6484):1346–1352.
18. Hollenstein K, et al. (2014) Insights into the structure of class B GPCRs. *Trends Pharmacol. Sci.* 35(1):12–22.
19. Nijmeijer S, Wolf S, Ernst OP, de Graaf C (2016) 7TM domain structure of adhesion GPCRs in *Handb. Exp. Pharmacol.* (Springer New York LLC) Vol. 234, pp. 43–66.
20. Hansen LH, et al. (1996) The Gly40Ser mutation in the human glucagon receptor gene associated with NIDDM results in a receptor with reduced sensitivity to glucagon. *Diabetes* 45(6):725–730.
21. Roberts DJ, Vertongen P, Waelbroeck M (2011) Analysis of the glucagon receptor first extracellular loop by the substituted cysteine accessibility method. *Peptides* 32(8):1593–1599.
22. Cascieri MA, et al. (1999) Characterization of a novel, non-peptidyl antagonist of the human glucagon receptor. *J. Biol. Chem.* 274(13):8694–8697.
23. Koth CM, et al. (2012) Molecular basis for negative regulation of the glucagon receptor. *Proc. Natl. Acad. Sci.* 109(36):14393–14398.
24. Assil-Kishawi I, Abou-Samra AB (2002) Sauvagine cross-links to the second extracellular loop of the corticotropin-releasing factor type 1 receptor. *J. Biol. Chem.* 277(36):32558–32561.
25. Prévost M, et al. (2010) Mutational and cysteine scanning analysis of the glucagon receptor N-terminal domain. *J. Biol. Chem.* 285(40):30951–30958.
26. Zhao LH, et al. (2016) Differential requirement of the extracellular domain in activation of class B G protein-coupled receptors. *J. Biol. Chem.* 291(29):15119–15130.
27. Yang D, et al. (2016) Structural determinants of binding the seven-transmembrane domain of the glucagon-like peptide-1 receptor (GLP-1R). *J. Biol. Chem.* 291(25):12991–13004.
28. Hollenstein K, et al. (2013) Structure of class B GPCR corticotropin-releasing factor receptor 1. *Nature* 499(7459):438–443.
29. Dore AS, et al. (2017) Decoding corticotropin-releasing factor receptor type 1 crystal structures. *Curr. Mol. Pharmacol.* 10(4).
30. Liang YL, et al. (2017) Phase-plate cryo-EM structure of a class B GPCR-G-protein complex. *Nature* 546(7656):118–123.
31. Song G, et al. (2017) Human GLP-1 receptor transmembrane domain structure in complex with allosteric modulators. *Nature* 546(7657):312–315.
32. Zhang Y, et al. (2017) Cryo-EM structure of the activated GLP-1 receptor in complex with a G protein. *Nature* 546(7657):248–253.
33. Ehrenmann J, et al. (2018) High-resolution crystal structure of parathyroid hormone 1 receptor in complex with a peptide agonist. *Nat. Struct. Mol. Biol.* 25(12):1086–1092.
34. Liang YL, et al. (2018) Cryo-EM structure of the active, Gs-protein complexed, human CGRP receptor. *Nature* 561(7724):492–497.
35. dal Maso E, et al. (2019) The molecular control of calcitonin receptor signaling. *ACS Pharmacol. Transl. Sci.* 2(1):31–51.
36. Xu Y, et al. (2019) Mutagenesis facilitated crystallization of GLP-1R. *IUCrJ* 6(6):996–1006.

37. Zhao LH, et al. (2019) Structure and dynamics of the active human parathyroid hormone receptor-1. *Science (80-.).* 364(6436):148–153.
38. Liang YLL, et al. (2020) Toward a structural understanding of class B GPCR peptide binding and activation. *Mol. Cell* 77(3):656–668.e5.
39. Ma S, et al. (2020) Molecular basis for hormone recognition and activation of corticotropin-releasing factor receptors. *Mol. Cell* 77(3):669–680.e4.
